# Medical care and biomarker-based assessment of mortality in two cohorts of patients with chronic coronary syndrome 10 years apart

**DOI:** 10.1186/s12872-023-03469-4

**Published:** 2023-08-29

**Authors:** Martin Rehm, Andrea Jaensch, Ben Schöttker, Ute Mons, Harry Hahmann, Wolfgang Koenig, Hermann Brenner, Dietrich Rothenbacher

**Affiliations:** 1 Institute of Epidemiology and Medical Biometry, Ulm University, Ulm, Germany; 2 Division of Clinical Epidemiology and Aging Research, German Cancer Research Center (DKFZ), Heidelberg, Germany; 3 Department of Cardiology, Faculty of Medicine and University Hospital Cologne, University of Cologne, Cologne, Germany; 4 Klinik Schwabenland, Isny-Neutrauchburg, Isny, Germany; 5 Deutsches Herzzentrum München, Technische Universität München, Munich, Germany; 6 German Centre for Cardiovascular Research (DZHK), partner site Munich Heart Alliance, Munich, Germany; 7 Division of Preventive Oncology, German Cancer Research Center (DKFZ) and National Center for Tumour Diseases (NCT), Heidelberg, Germany; 8 German Cancer Consortium (DKTK), German Cancer Research Center (DKFZ), Heidelberg, Germany

**Keywords:** Chronic coronary syndrome, Cardiac rehabilitation, Risk factors, Biomarkers, Pharmacological treatment, Mortality

## Abstract

**Background:**

This study aimed to describe the characteristics and mortality of two cohorts of patients with chronic coronary syndrome (CCS) recruited with identical study designs in the same rehabilitation clinics but approximately 10 years apart.

**Methods:**

The KAROLA cohorts included patients with CCS participating in an inpatient cardiac rehabilitation programme in Germany (KAROLA-I: years 1999/2000, KAROLA-II: 2009–2011). Blood samples and information on sociodemographic factors, lifestyle, and medical treatment were collected at baseline, at the end of rehabilitation, and after one year of follow-up. A biomarker-based risk model (ABC-CHD model) and Cox regression analysis were used to evaluate cardiovascular (CV) and non-CV mortality risk.

**Results:**

We included 1130 patients from KAROLA-I (mean age 58.7 years, 84.4% men) and 860 from KAROLA-II (mean age 60.4 years, 83.4% men). Patients in the KAROLA-I cohort had significantly higher concentrations of CV biomarkers and fewer patients were taking CV medications, except for statins. The biomarker-based ABC-CHD model provided a higher estimate of CV death risk for patients in the KAROLA-I cohort (median 3-year risk, 3.8%) than for patients in the KAROLA-II cohort (median 3-year risk, 2.7%, p-value for difference < 0.001). After 10 years of follow-up, 91 (8.1%) patients in KAROLA-I and 45 (5.2%) in KAROLA-II had died from a CV event.

**Conclusions:**

Advances in disease management over the past 20 years may have led to modest improvements in pharmacological treatment during cardiac rehabilitation and long-term outpatient care for patients with CCS. However, modifiable risk factors such as obesity have increased in the more recent cohort and should be targeted to further improve the prognosis of these patients.

## Background

Cardiovascular diseases (CVD) remain the most common cause of death in Germany and worldwide [1]. However, the number of deaths due to CVD in Germany has declined continuously over the past 20 years [2]. While deaths due to diseases of the circulatory system accounted for 48% of the total number of deaths in Germany in 1999, this proportion went down to 42% in 2009 and 35% in 2019 [3].

The reasons for this decline are manifold. An important starting point was identifying the significant risk factors for CVD, including smoking, high cholesterol, hypertension, physical inactivity, and diabetes [4]. Translating these scientific findings into prevention targets, educational campaigns, and clinical treatment strategies has improved individual cardiovascular (CV) prognosis. Identifying factors influencing the further development of CVD was also an important prerequisite for the progressive development of therapeutic measures such as improved blood pressure control or lipid management by cholesterol-lowering drugs [4]. Tertiary prevention programmes for patients with chronic coronary syndrome (CCS) are also under constant revision and improvement [5].

Several tools have been developed to predict the risk of recurrent CV events. Earlier tools included socioeconomic information, lifestyle-associated risk factors, and clinical characteristics. In addition, the discovery of biomarkers that can be used for monitoring biological systems or for risk stratification has also gained importance for predicting the occurrence of CVD and has proven to be a valuable approach for risk identification and prevention at earlier stages of the disease process [6].

The ABC-CHD risk model incorporates novel biomarkers such as N-terminal pro-B-natriuretic peptide (NT-proBNP) and cardiac troponin T (hs-cTnT) in addition to conventional factors in a unifying risk score and is considered good clinical practice for assessing the risk profile for CV mortality in patients with CCS [5, 7]. However, data from the tertiary prevention setting, including different comparison periods, are scarce.

This study aimed to describe the characteristics and mortality of two cohorts of patients with CCS recruited with identical study designs in the same rehabilitation clinics but approximately 10 years apart. In particular, the aim was to investigate potential differences in sociodemographic and lifestyle risk factors, derive possible differences in disease management and pharmacological treatment, and quantify the risk of subsequent short-term CV mortality using a biomarker-based risk model. In addition, CV and non-CV mortality were further investigated using a conventional survival analysis approach.

## Methods

### Study population and study design

The prospective KAROLA cohort studies included patients with chronic coronary syndrome (CCS) who participated in an inpatient cardiac rehabilitation programme at one of two contributing rehabilitation clinics in Germany (Schwabenland-Klinik, Isny, and Klinik am Südpark, Bad Nauheim) within three months of their first acute event or coronary bypass surgery. In Germany, all patients discharged from an acute hospital after an acute coronary syndrome or coronary bypass surgery are offered a comprehensive inpatient rehabilitation programme.

KAROLA-I included patients aged 30–70 years who were admitted for rehabilitation between January 1999 and May 2000. Of all eligible patients admitted to the rehabilitation clinic during recruitment, 58% agreed to participate (*n* = 1206). A detailed description of the study methods has been previously published [8]. Although we recruited patients in only two such inpatient rehabilitation hospitals (one in southern Germany and the other in central Germany), these specialized hospitals serve a large geographic area with a radius of up to 200 km, so patients were referred from a large number of different acute care hospitals and therefore represent a considerable fraction of the target population. All patients provided written informed consent. The study was approved by the ethics committees of the Universities of Ulm (no. 186/98) and Heidelberg and by the medical associations of the states of Baden-Wuerttemberg and Hessen.

KAROLA-II is a second cohort study that included patients aged 30–75 years admitted for rehabilitation at the same two collaborating rehabilitation clinics between September 2009 and June 2011. In this context, the assignment criteria for inpatient rehabilitation have not changed since the enrolment of the first cohort. The participation rate was also 58% (*n* = 1149). Again, all patients provided written informed consent. The study was approved by the ethics committee of the University of Heidelberg (no. 351/2001). Apart from the higher upper age limit for the inclusion criteria, the study design and methods are identical in KAROLA-I and KAROLA-II.

**Fig. 1:**
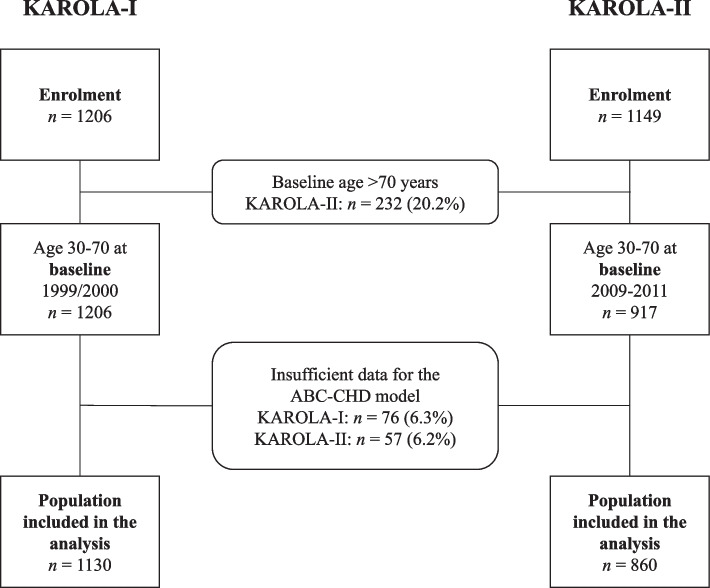
Study flowchart

For this comparative analysis, we restricted both cohorts to patients who were between 30 and 70 years of age at baseline and had sufficient data for the ABC-CHD model (Fig. 1).

### Data collection

At the beginning of the inpatient rehabilitation programme, all patients completed a standardised questionnaire that included sociodemographic information and medical history. Medication at discharge from the rehabilitation clinic (therapy recommendation) was requested from the attending physician. Treatment with high-potency statins was defined as the prescription of atorvastatin or rosuvastatin. In addition, information on physical activity and statin treatment was collected during the postal follow-up of patients one year after discharge.

Fasting blood samples were taken at the end of inpatient cardiac rehabilitation. Serum samples were centrifuged within 2 h. Samples were stored at -20 °C for up to 1 month and then transferred to a -80 °C freezer until analysis.

Blood lipids were determined at baseline using routine methods in a central laboratory. Cystatin C was measured by immunonephelometry on a Behring Nephelometer II (Dade-Behring, Marburg, Germany). High-sensitivity C-reactive protein (hs-CRP) was determined on the same device using latex-enhanced nephelometry. High-sensitivity cardiac troponin T (hs-cTnT) measurements were performed on an Elecsys 2010 platform (Roche Diagnostics, Penzberg, Germany). In parallel, high-sensitivity cardiac troponin I (hs-cTnI) was determined on an Abbott ARCHITECT i1000 platform. N-terminal pro-B-natriuretic peptide (NT-proBNP) was measured by electrochemiluminescence on an Elecsys 170 (Roche Diagnostics, Penzberg, Germany). Finally, GDF-15 serum concentrations were measured by Electrochemiluminescence (ECLIA, Cobas Elecsys 411, Roche Diagnostics, Penzberg, Germany). Cardiac marker measurements for KAROLA-I were performed between 2003 (NT-proBNP) and 2010 (troponins) and for KAROLA-II in 2016 (respectively) from serum stored at -80º Celsius. All laboratory measurements were performed in a blinded fashion.

### Follow-up for mortality

Registration offices were contacted to obtain survival status and the date of death if the patient had died. Death certificates were obtained from local health departments, and the main cause of death was coded according to the International Classification of Diseases (ICD). CV mortality was defined as CVD (ICD-9 items 390–459; ICD-10 items I00-I99 and R57.0) as the main cause of death, and non-CV mortality as anything that had another main cause of death. Follow-up time was calculated in days, beginning with the date of admission to the rehabilitation clinic and ending with the date of death. Patients who did not die within 10 years of follow-up were censored at the last contact (last sign of life) or after 10 years (right truncation after 3652 days) to ensure comparability between cohorts.

### Statistical analysis

Baseline characteristics of the study population are reported as absolute and relative frequencies for categorical variables and means and standard deviations for continuous variables. Generalised linear models were used to test various concentrations of CV risk markers at baseline for differences between study cohorts when adjusted for age and sex. In addition, a biomarker-based risk model (ABC-CHD model) was used to predict CV mortality risk for each patient after 1, 2, and 3 years of follow-up, as suggested by Lindholm et al. [7]. The duration of follow-up in the biomarker sub-study of the STABILITY (Stabilization of Atherosclerotic Plaque by Initiation of Darapladib Therapy) trial, on whose data the prediction model was developed, was only three years, so longer survival estimates are not possible with the model. The variables used in the ABC-CHD model were age, current smoking, diabetes mellitus, peripheral arterial disease, hs-cTnT, NT-proBNP, and LDL-cholesterol at baseline. In addition, a Cox proportional hazards model adjusted for established covariates was used to estimate the association with CV mortality during the 10-year follow-up period of KAROLA-II compared to KAROLA-I. The risk estimates for statin medication were also derived from the latter model in a secondary analysis. Furthermore, cumulative incidence curves for CV and non-CV deaths were calculated as competing risks using the Aalen-Johansen estimator during the 10 years of follow-up [9]. The parameters used to compute an estimate of a survival curve for censored data using the Aalen-Johansen estimator are the follow up time (up to 3652 days), the event indicator (Censor, CV mortality or non-CV mortality) and the cohort indicator (KAROLA-I or KAROLA-II). Statistical analysis was performed with R version 4.1.2 (R Foundation for Statistical Computing, Vienna, Austria).

## Results

### Patient characteristics

Table 1 summarises the baseline characteristics of the 1130 patients in KAROLA-I (age range 30–70 years, mean age 58.7 years, 84.4% men) and the 860 patients in KAROLA-II (age range 30–70 years, mean age 60.4 years, 83.4% men). The proportion of obese patients with a body mass index of 30.0 or greater kg/m^2^ was higher in KAROLA-II (15.6% in KAROLA-I compared to 24.3% in KAROLA-II), and the same was true for the prevalence of current smokers (5.3% in KAROLA-I vs 7.4% in KAROLA-II). In KAROLA-I, more patients had less than 10 years of school education than in KAROLA-II (59.6% vs 48.0%). The proportion of patients with a history of myocardial infarction (MI) was higher in KAROLA-I compared to KAROLA-II (59.1% vs 50.1%). In the KAROLA-I cohort, 38.5% had undergone percutaneous coronary intervention (PCI), whereas 46.9% had received coronary artery bypass grafting (CABG). The frequency distribution for choice of revascularisation procedure was quite different in KAROLA-II, where 59.1% had received a PCI, and only 35.2% CABG. Other demographic and clinical characteristics were very similar in both cohorts.

**Table 1:** Baseline characteristics of the study population

	KAROLA-I	KAROLA-II	
	Year 1999/2000	Year 2009–2011	
Characteristic	(*n* = 1130)	(*n* = 860)	*p*-value
Age (years)	58.7 (8.1)	60.4 (8.0)	< 0.001^a^
Men, n (%)	954 (84.4)	717 (83.4)	0.567^b^
Body mass index (BMI, kg/m^2^)	26.9 (3.2)	27.7 (3.8)	< 0.001^a^
Obese (BMI ≥ 30.0 kg/m^2^), n (%)	176 (15.6)	209 (24.3)	< 0.001^b^
School education < 10 years, n (%)	673 (59.6)	413 (48.0)	< 0.001^b^
Physical activity (hours/week)			
Within past year	6.5 (7.6)	6.5 (6.8)	0.979^a^
At 1-year follow-up	7.3 (6.5)	6.7 (7.2)	0.123^a^
Smoking status, n (%)			0.149^b^
Never	354 (31.3)	265 (30.8)	
Former	716 (63.4)	531 (61.7)	
Current	60 (5.3)	64 (7.4)	
History of diabetes mellitus, n (%)	197 (17.4)	165 (19.2)	0.345^b^
History of hypertension, n (%)	621 (55.0)	501 (58.3)	0.069^b^
History of myocardial infarction, n (%)	668 (59.1)	431 (50.1)	< 0.001^b^
History of heart failure, n (%)	133 (11.8)	94 (10.9)	0.029^b^
History of peripheral arterial disease, n (%)	47 (4.2)	32 (3.7)	0.704^b^
Clinical score (angiographic evaluation), n (%)			0.027^b^
0 vessel disease	16 (1.4)	4 (0.5)	
1 vessel disease	281 (24.9)	212 (24.7)	
2 vessel disease	298 (26.4)	222 (25.8)	
3 vessel disease	478 (42.3)	354 (41.2)	
Unknown	57 (5.0)	68 (7.9)	
Heart rate (beats/min)	73.2 (9.8)	70.0 (9.7)	< 0.001^a^
Systolic blood pressure (mmHg)	119.8 (15.5)	119.8 (13.6)	0.979^a^
Percutaneous coronary intervention, n (%)	435 (38.5)	508 (59.1)	< 0.001^b^
Coronary artery bypass grafting, n (%)	530 (46.9)	303 (35.2)	< 0.001^b^

Values are reported as absolute and relative frequencies for categorical variables and means (standard deviations) for continuous variables

^a^One-way ANOVA

^b^Chi-square test

### Concentrations of CV risk markers

Comparing CV risk markers between the two cohorts revealed significantly higher age- and sex-adjusted concentrations of all considered biomarkers in KAROLA-I vs KAROLA-II (Table 2). This pattern was identical when stratified by the history of MI (data not shown).

**Table 2:** Age- and sex-adjusted baseline concentrations of cardiovascular risk markers

	**KAROLA-I**		**KAROLA-II**		
	**Year 1999/2000**		**Year 2009–2011**		
	**(*n* = 1130)**		**(*n* = 860)**		
**Marker**	**n**	**Geometric Mean**	**n**	**Geometric Mean**	***p*-value**^**a**^
Total cholesterol (mg/dL)	1130	166.6	859	148.2	< 0.001
LDL-cholesterol (mg/dL)	1130	96.8	860	85.8	< 0.001
Cystatin C (mg/L)	1129	1.1	860	1.0	< 0.001
hs-CRP (mg/L)	1129	3.5	860	2.2	< 0.001
hs-cTnT (ng/L)	1130	13.9	860	11.5	< 0.001
hs-cTnI (ng/L)	1112	16.5	859	11.6	< 0.001
NT-proBNP (ng/L)	1130	577.4	860	390.4	< 0.001
GDF-15 (ng/L)	1128	1314.5	849	1164.0	< 0.001

Values are reported as age- and sex-adjusted geometric means

^a^Generalised linear model

### Medication upon discharge from the rehabilitation clinic

Table 3 shows the medication at discharge from the rehabilitation clinic for both cohorts. Except for statins, which showed a similar proportion in both cohorts, the proportion of patients on CV medications was significantly higher in KAROLA-II than in KAROLA-I. While in KAROLA-I, high-potency statins (atorvastatin and rosuvastatin) were recommended in 31.2% of patients at discharge, this proportion was only 9.4% in KAROLA-II. However, in the latter cohort, a significant increase was seen for simvastatin, ezetimibe, and a combination of both drugs. The picture was very similar when looking at patient-reported medication at 1-year follow-up: 32.8% of patients in KAROLA-I and 8.1% in KAROLA-II reported taking high-potency statins (data not included in the table).

**Table 3:** Medication upon discharge from the rehabilitation clinic

	KAROLA-I	KAROLA-II	
	Year 1999/2000	Year 2009–2011	
Active ingredient group	(*n* = 1130)	(*n* = 860)	*p*-value^**a**^
Acetylsalicylic acid, n (%)	985 (87.2)	838 (97.4)	< 0.001
Diuretics, n (%)	233 (20.6)	250 (29.1)	< 0.001
Beta-blocker, n (%)	980 (86.7)	792 (92.1)	< 0.001
Calcium channel blocker, n (%)	92 (8.1)	112 (13.0)	< 0.001
ACE inhibitors, n (%)	566 (50.1)	541 (62.9)	< 0.001
Statins, n (%)	859 (76.0)	639 (74.3)	0.409
High-potency statins^b^	352 (31.2)	81 (9.4)	< 0.001
Moderate-potency statins	391 (34.6)	536 (62.3)	< 0.001
Ezetimibe^c^	0 (0.0)	187 (21.7)	< 0.001

Values are reported as absolute and relative frequencies

^a^Chi-square test

^b^Atorvastatin and Rosuvastatin

^c^Including *n* = 173 patients receiving the drug INEGY (combination of Ezetimibe and Simvastatin)

### Biomarker-based estimates of CV death risk and survival status

Biomarker-based estimates of CV death risk based on the (short-term) ABC-CHD model and survival status after 10 years of follow-up are reported in Table 4. The ABC-CHD model provided a higher estimate of CV death risk for patients in KAROLA-I (median 3-year risk, 3.8%) than for patients in KAROLA-II (median 3-year risk, 2.7%, *p* < 0.001).

**Table 4:** Biomarker-based estimates of cardiovascular death risk and survival status

	KAROLA-I	KAROLA-II	
	Year 1999/2000	Year 2009–2011	
Characteristic	(*n* = 1130)	(*n* = 860)	*p*-value
**ABC-CHD model**^a^			
Estimated CV death risk (%)			
Median 1-year risk	1.1	0.8	< 0.001^b^
Median 2-year risk	2.4	1.7	< 0.001^b^
Median 3-year risk	3.8	2.7	< 0.001^b^
**Follow-up for mortality (10 years)**			
Censored, n (%)	970 (85.8)	756 (87.9)	
CV mortality			
n (%)	91 (8.1)	45 (5.2)	
Rate per 1000 p-y (95% CI)	8.7 (7.1–10.6)	5.9 (4.4–7.9)	
Cox-model 1, HR (95% CI)	1.00 (reference)	0.63 (0.44–0.91)	0.012^c^
Cox-model 2, HR (95% CI)	1.00 (reference)	0.77 (0.53–1.14)	0.190^c^
Non-CV mortality			
n (%)	69 (6.1)	59 (6.9)	
Rate per 1000 p-y (95% CI)	6.6 (5.2–8.3)	7.7 (6.0–10.0)	
Cox-model 1, HR (95% CI)	1.00 (reference)	1.05 (0.74–1.49)	0.792^c^
Cox-model 2, HR (95% CI)	1.00 (reference)	1.01 (0.70–1.47)	0.956^c^

*CV* Cardiovascular, *HR* Hazard Ratio

^a^The variables used in the ABC-CHD model were age, current smoking, history of diabetes mellitus, peripheral arterial disease, hs-cTnT, NT-proBNP, and LDL-cholesterol. The ABC-CHD model is based on the 3-year biomarker substudy of the STABILITY (Stabilization of Atherosclerotic Plaque by Initiation of Darapladib Therapy) trial.

Cox regression analyses were performed after adjustment for age and sex (model 1) and additionally adjusted for body mass index, systolic blood pressure, current smoking, history of diabetes mellitus, LDL-cholesterol, and established markers of CV-risk (NT-proBNP and hs-cTnT) (model 2).

^b^Kruskal-Wallis Rank Sum test.

^c^Cox-model.

After 10 years of follow-up, 91 (8.1%) patients in KAROLA-I (8.7 deaths per 1000 person‐years) and 45 (5.2%) in KAROLA-II (5.9 deaths per 1000 person‐years) had died because of CV events. Patients in the KAROLA-II cohort had a lower hazard ratio (HR) for CV mortality when analysed in an age- and sex-adjusted Cox proportional hazards model (HR 0.63 (95% confidence interval (CI) 0.44–0.91)). The point estimate increased to a HR of 0.77 when additional established risk factors and biomarkers were added, but the 95% confidence interval included the null effect (additional adjustment for schooling, history of MI, and CABG or PCI yielded similar results). However, non-CV mortality was similar in both cohorts. The proportion of patients who died due to other causes was 6.1% and 6.9%, respectively.

When statin medication upon discharge was included as an exposure variable in the Cox model that included age, sex, biomarkers, and clinical variables for the combined analysis with both cohorts, the HR for CV mortality was 0.54 (95% CI 0.31–0.93) in patients taking high-potency statins compared with patients not on statin medication (details in Table 5).

**Table 5:** Association of statin medication with CV mortality during the 10-year follow-up

	Fatal CV	Patients	CV	Rate per	Hazard Ratio^**a**^	
	Events	at Risk	Mortality	1000 py	(95% CI)	*p*-value^**a**^
**Statin medication**^b^						
None	46	492	9.3%	10.3	1.00 (Reference)	
Low/Moderate Potency	70	1065	6.6%	7.3	0.87 (0.59–1.28)	0.466
High Potency^c^	20	433	4.6%	4.9	0.54 (0.31–0.93)	0.027
Overall^d^	136	1990	6.8%	7.5		

^a^Cox-model adjusted for cohort, age, sex, body mass index, systolic blood pressure, current smoking, history of diabetes mellitus, LDL-cholesterol, and established markers of CV-risk (NT-proBNP and hs-cTnT)

^b^Statin medication upon discharge from the rehabilitation clinic

^c^Atorvastatin and Rosuvastatin. ^d^Both KAROLA cohorts combined

**Fig. 2:**
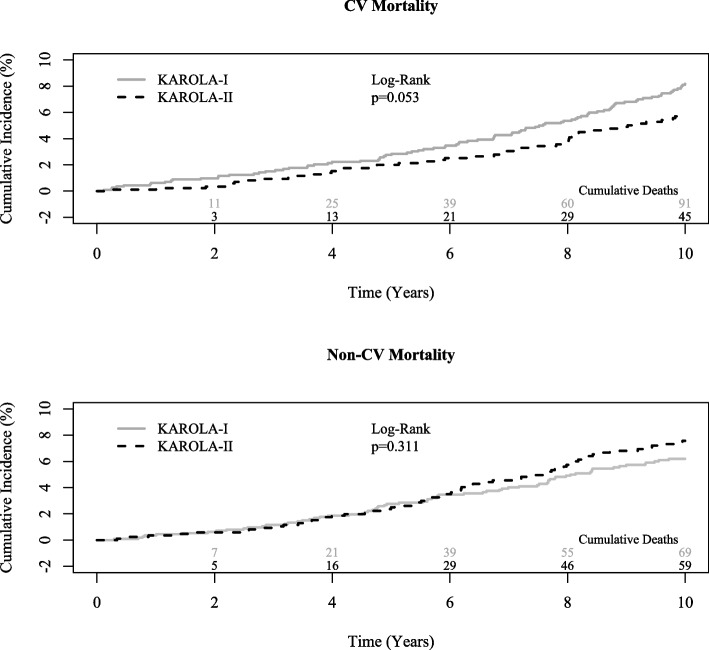
Cumulative incidence curves for competing mortality events in KAROLA-I (*n* = 1130) and KAROLA-II (*n* = 860) (cumulative deaths over time at the bottom of the graphs)

The cumulative incidence curves with CV and non-CV mortality as competing risks are shown in Fig. 2. CV mortality curves differed by cohort, with a slightly higher incidence in the KAROLA-I cohort, although the log-rank test did not yield formal statistical significance (*p* = 0.053). In contrast, the non-CV mortality curves showed a higher incidence in KAROLA-II with advanced follow-up time.

## Discussion

In this study, patients with CCS who had participated in an inpatient cardiac rehabilitation programme in 1999 or 2000 were less likely to have been treated with specific CV medication, except for statins, and had higher baseline concentrations of CV risk biomarkers than patients who participated in such a programme approximately 10 years later. Notably, this resulted in higher biomarker-based estimates of CV death risk from the (short-term) ABC-CHD model for the earlier cohort, an observation that was no longer statistically significant when 10-year CV mortality was considered in a fully adjusted model. In contrast, with regard to non-CV mortality, there was only a very slight difference. However, risk factors such as obesity were more prevalent in the more recent cohort and should be targeted to further improve the long-term prognosis of these patients. A clear difference was also seen in the prescription of statins, also in combination with a non-statin, in very high-risk patients over the last two decades.

Cardiac rehabilitation is an intervention to improve the functional capacity and health-related quality of life of patients with heart disease and is considered a clinically effective intervention for patients after ACS or coronary revascularisation [10, 11]. According to the current 2019 ESC guidelines, the overall management of CCS aims to reduce symptoms and improve prognosis through appropriate CVD medications and interventions, as well as control risk factors such as obesity and unhealthy lifestyle habits like smoking, reduce sedentary behaviour, and increase physical activity [5]. In addition to evidence-based drug therapy, promotion of treatment adherence, behavioural counselling, and assistance in controlling lifestyle risk factors play an essential role [5]. Better control of risk factors through lifestyle changes and adherence to preventive pharmacotherapy are important aims for improving prognosis and long-term survival and may partly explain the significant disparities in CV mortality across European countries [12].

However, achieving the secondary prevention targets recommended in the respective guidelines is often suboptimal and hampered by many barriers. For example, the EUROASPIRE study carried out in 2016/2017 in 27 countries showed that risk factors assessed according to the 2016 ESC guideline targets were still not under control in a substantial proportion of patients. 55% of smokers from the baseline survey still had not quit more than 6 months later, 38% were obese, 66% continued to take little exercise, and 42% and 71%, respectively, did not reach target levels for blood pressure and LDL-cholesterol, although there was some progress compared with previous surveys [13]. Our study population also showed substantial improvements in all CV biomarkers, indicating advances in pharmacological and interventional approaches, yet the prevalence of traditional risk factors such as cigarette smoking and body weight control showed no improvement over these 10 years. Smoking, in particular, is an important target for intervention, and smoking cessation shows benefits in terms of secondary vascular disease after only a few years [14].

The lower proportion of patients with a history of MI and the lower biomarker concentrations in the more recent KAROLA-II cohort suggest that the detection and monitoring of CV risk factors improved significantly over the observed decade between these cohorts. However, our data do not show improvements in all of these areas, and further efforts appear worthwhile. This is also true for body weight, which increased over 10 years in this population.

In the development of the ratio of bypass surgery to PCI, our cohorts show a clear trend towards the less invasive intervention. This is a trend that was observed nationwide during this period [15]. Advances in surgical procedures and other improvements, such as dual platelet inhibition after drug-eluting stent implantation, may also have contributed to a reduction in CV mortality.

The significantly higher proportion of patients in KAROLA-II taking CV medications suggests a greater focus on appropriate medical secondary prevention therapy (e.g., aspirin, beta-blocker, ACE inhibitors, diuretics) and may have contributed at least in part to the observed lower CV death risk. It is interesting that statins, which represent the most important antiatherosclerotic medication, were not prescribed more frequently but that a substantial shift towards ezetimibe and its combination with simvastatin for achieving LDL target values was already observed in 2009–2011 (which were < 100 mg/dl in the ESC guidelines valid at the time) [16].

High-potency statins, which nowadays are strongly recommended by cardiovascular societies such as the American Heart Association (AHA), the European Society of Cardiology (ESC), and the German Cardiac Society (DGK), indeed showed significant protection in our analyses. However, atorvastatin was patent-protected until 2012 and rosuvastatin until 2020 and was therefore not prescribed in Germany for patients with statutory health insurance. The IMPROVE-IT trial was the first study to show the benefit of combining a non-statin (ezetimibe) with simvastatin in high-risk patients following ACS, and this treatment strategy was subsequently included in the guidelines [17]. The recent observational DA VINCI study noted the persistent discrepancy between current guideline recommendations and recent clinical practice for lipid management in Europe [18]. The use of high-potency statins decreased from 53% in 2016 to only 30% in 2019. In the context of this study in Germany, only 16% received high-intensity statin therapy in primary and secondary prevention [19]. According to recent European guidelines, patients with established CVD are considered very high-risk and besides lifestyle interventions, statins are recommended for all patients with CCS [20].

We also observed substantial improvements in biomarker-based estimates of risk for (short-term) CV death. This suggests improvements in targeting established risk factors and earlier disease detection and intervention. Other emerging CV risk factors, such as air pollution and noise leading to sleep disturbances, may be on the rise, partially reversing the gains in controlling traditional risk factors [21–23]. This may also potentially explain the time trends observed in 20 Western European countries in the Global Burden of Disease study, which showed a decline in ischemic heart disease indicators and mortality over the past 30 years, but already showed a levelling off in recent years [24].

One limitation of the present study is that only patients referred for inpatient cardiac rehabilitation and willing to participate were included, so critically ill patients may be underrepresented. During the recruitment period, the criteria for admission to an inpatient rehabilitation facility did not change considerably, but the capacity of the rehabilitation system to care for more severely ill patients increased. Therefore, we assume that older patients were more frequently admitted for inpatient rehabilitation in the recent KAROLA cohort, which is reflected in a higher mean age in KAROLA-II. A second limitation is that information on medication adherence was not available, so that adherence to medication—an important factor for the long-term effect of treatment—could not be taken into account.

Among the strengths of the current study is the detailed characterisation of the study populations at baseline, which provides information on established risk factors for death and CVD. Other strengths include that biomarker concentrations could be measured with high-sensitivity, high-quality assays in the same laboratory and that the same study design and methods were used to recruit these cohorts, which are approximately 10 years apart, resulting in excellent comparability between these two cohorts.

Comparing these two cohorts suggests considerable progress in controlling underlying risk factors and medication management at discharge from inpatient cardiac rehabilitation within 10 years. However, further research and development are needed to better understand disease-causing factors and interventions to improve patient care, which will ultimately further improve the health of CV patients.

## Conclusions

Advances in disease management over the past 20 years may have led to modest improvements in pharmacological treatment during cardiac rehabilitation and long-term outpatient care for patients with CCS. However, modifiable risk factors such as obesity have increased in the more recent cohort and should be targeted to further improve the prognosis of these patients.

## Data Availability

The datasets used and/or analysed during the current study are available from the corresponding author on reasonable request.

## References

[1] Piepoli MF, Hoes AW, Agewall S, Albus C, Brotons C, Catapano AL (2016). 2016 European Guidelines on cardiovascular disease prevention in clinical practice: The Sixth Joint Task Force of the European Society of Cardiology and Other Societies on Cardiovascular Disease Prevention in Clinical Practice (constituted by representatives of 10 societies and by invited experts)Developed with the special contribution of the European Association for Cardiovascular Prevention & Rehabilitation (EACPR). Eur Heart J.

[2] Moran AE, Forouzanfar MH, Roth GA, Mensah GA, Ezzati M, Murray CJL (2014). Temporal trends in ischemic heart disease mortality in 21 world regions, 1980 to 2010: the Global Burden of Disease 2010 study. Circulation.

[3] Statistisches Bundesamt Deutschland - GENESIS-Online. 2021. https://www-genesis.destatis.de/genesis/online?operation=statistic&levelindex=0&levelid=1625476397134&code=23211#abreadcrumb. Accessed 5 Jul 2022.

[4] Libby P (2021). The changing landscape of atherosclerosis. Nature.

[5] Knuuti J, Wijns W, Saraste A, Capodanno D, Barbato E, Funck-Brentano C (2020). 2019 ESC Guidelines for the diagnosis and management of chronic coronary syndromes: The Task Force for the diagnosis and management of chronic coronary syndromes of the European Society of Cardiology (ESC). Eur Heart J.

[6] Rossello X, Dorresteijn JA, Janssen A, Lambrinou E, Scherrenberg M, Bonnefoy-Cudraz E (2019). Risk prediction tools in cardiovascular disease prevention: A report from the ESC Prevention of CVD Programme led by the European Association of Preventive Cardiology (EAPC) in collaboration with the Acute Cardiovascular Care Association (ACCA) and the Association of Cardiovascular Nursing and Allied Professions (ACNAP). Eur J Prev Cardiol.

[7] Lindholm D, Lindbäck J, Armstrong PW, Budaj A, Cannon CP, Granger CB (2017). Biomarker-Based Risk Model to Predict Cardiovascular Mortality in Patients With Stable Coronary Disease. J Am Coll Cardiol.

[8] Rothenbacher D, Hahmann H, Wüsten B, Koenig W, Brenner H (2007). Symptoms of anxiety and depression in patients with stable coronary heart disease: prognostic value and consideration of pathogenetic links. Eur J Cardiovasc Prev Rehabil.

[9] Aalen OO, Johansen S (1978). An Empirical Transition Matrix for Non-Homogeneous Markov Chains Based on Censored Observations. Scand J Stat.

[10] Rauch B, Davos CH, Doherty P, Saure D, Metzendorf M-I, Salzwedel A (2016). The prognostic effect of cardiac rehabilitation in the era of acute revascularisation and statin therapy: A systematic review and meta-analysis of randomized and non-randomized studies – The Cardiac Rehabilitation Outcome Study (CROS). Eur J Prev Cardiol.

[11] de Vries H, Kemps HMC, van Engen-Verheul MM, Kraaijenhagen RA, Peek N (2015). Cardiac rehabilitation and survival in a large representative community cohort of Dutch patients. Eur Heart J.

[12] Hartley A, Marshall DC, Salciccioli JD, Sikkel MB, Maruthappu M, Shalhoub J (2016). Trends in mortality from ischemic heart disease and cerebrovascular disease in Europe: 1980 to 2009. Circulation.

[13] Kotseva K, De Backer G, De Bacquer D, Rydén L, Hoes A, Grobbee D (2019). Lifestyle and impact on cardiovascular risk factor control in coronary patients across 27 countries: results from the European society of cardiology ESC-EORP EUROASPIRE V registry. Eur J Prev Cardiol.

[14] Twardella D, Rothenbacher D, Hahmann H, Wüsten B, Brenner H (2006). The underestimated impact of smoking and smoking cessation on the risk of secondary cardiovascular disease events in patients with stable coronary heart disease: prospective cohort study. J Am Coll Cardiol.

[15] Deutsche Herzstiftung. Deutscher Herzbericht 2020. https://www.herzstiftung.de/e-paper/#70. Accessed 16 Oct 2022.

[16] Reiner Z, Catapano AL, De Backer G, Graham I, Taskinen M-R, European Association for Cardiovascular Prevention and Rehabilitation (2011). ESC/EAS Guidelines for the management of dyslipidaemias: the Task Force for the management of dyslipidaemias of the European Society of Cardiology (ESC) and the European Atherosclerosis Society (EAS). Eur Heart J.

[17] Eisen A, Cannon CP, Blazing MA, Bohula EA, Park J-G, Murphy SA (2016). The benefit of adding ezetimibe to statin therapy in patients with prior coronary artery bypass graft surgery and acute coronary syndrome in the IMPROVE-IT trial. Eur Heart J.

[18] Ray KK, Molemans B, Schoonen WM, Giovas P, Bray S, Kiru G (2021). EU-Wide Cross-Sectional Observational Study of Lipid-Modifying Therapy Use in Secondary and Primary Care: the DA VINCI study. Eur J Prev Cardiol.

[19] Gouni-Berthold I, Schaper F, Schatz U, Tabbert-Zitzler A, Fraass U, Sauer S (2022). Low-density lipoprotein cholesterol goal attainment in Germany: Results from the DA VINCI study. Atheroscler Plus.

[20] Mach F, Baigent C, Catapano AL, Koskinas KC, Casula M, Badimon L (2020). 2019 ESC/EAS Guidelines for the management of dyslipidaemias: lipid modification to reduce cardiovascular risk. Eur Heart J.

[21] Münzel T (2019). Up in the air: links between the environment and cardiovascular disease. Cardiovasc Res.

[22] Brook RD, Newby DE, Rajagopalan S (2017). Air Pollution and Cardiometabolic Disease: An Update and Call for Clinical Trials. Am J Hypertens.

[23] Drager LF, McEvoy RD, Barbe F, Lorenzi-Filho G, Redline S, INCOSACT Initiative (International Collaboration of Sleep Apnea Cardiovascular Trialists) (2017). Sleep Apnea and Cardiovascular Disease: Lessons From Recent Trials and Need for Team Science. Circulation.

[24] Vancheri F, Tate AR, Henein M, Backlund L, Donfrancesco C, Palmieri L (2022). Time trends in ischaemic heart disease incidence and mortality over three decades (1990–2019) in 20 Western European countries: systematic analysis of the Global Burden of Disease Study 2019. Eur J Prev Cardiol.

